# Synthesis and Optimization of Ti/Li/Al Ternary Layered Double Hydroxides for Efficient Photocatalytic Reduction of CO_2_ to CH_4_

**DOI:** 10.1038/s41598-019-41979-4

**Published:** 2019-04-04

**Authors:** Ting-Ting Kong, Jian Huang, Xin-Gang Jia, Wen-Zhen Wang, Yong Zhou

**Affiliations:** 1 College of Chemistry and Chemical Engineering, Xi’an Shiyou University, Xi’an, 710054 Shaanxi China; 2 Department of Physics, Nanjing University, Nanjing, 210039 Jiangsu China

## Abstract

A series of Ti/Li/Al ternary layered double hydroxides (TiLiAl-LDHs) with different Ti:Li:Al molar ratios were prepared by a coprecipitation method for photocatalytic CO_2_ reduction. It was demonstrated that the contents of anions between the layers of Ti/Li/Al-LDHs greatly determined the photocatalytic activity for CO_2_ reduction. With Ti:Li:Al molar ratios optimized to be 1:3:2, the largest contents of ${{\bf{CO}}}_{{\bf{3}}}^{{\bf{2}}}$^−^ anion and hydroxyl group were obtained for the Ti_1_Li_3_Al_2_-LDHs sample, which exhibited the highest photocatalytic activity for CO_2_ reduction, with CH_4_ production rate achieving 1.33 mmol h^−1^ g^−1^. Moreover, the theoretical calculations show that Ti_1_Li_3_Al_2_-LDHs is a p-type semiconductor with the narrowest band gap among all the obtained TiLiAl-LDHs. After calcined at high temperatures such as 700 °C, and the obtained TiLiAl-700 sample showed much increased photocatalytic activity for CO_2_ reduction, with CH_4_ production rate reaching about 1.59 mmol h^−1^ g^−1^. This calcination induced photocatalytic enhancement should be related to the cystal structure transformation from hydrotalcite to mixed oxides containing high reactive oxygen species for more efficient CO_2_ reduction.

## Introduction

The greenhouse effect caused by greenhouse gases has seriously affected people’s life and social development^1,2^. CO_2_ has been believed as a major greenhouse gas, and its emission controlling has become a key problem faced by our human society^3,4^. Physical adsorption and photocatalytic reduction have been considered to be the most promising methods for CO_2_ emission reduction^5,6^. However, photoreduction of CO_2_ is a complex and difficult reaction with a lot of carbon species produced^7^, the searching for high efficiency adsorbents and photocatalysts is the key priority to the technological breakthrough^8^. Until now, different kinds of semiconductors, including oxides, sulfides and nitrides, have been developed for photocatalytic CO_2_ reduction, and yet shown unsatisfying activity and selectivity^9^.

In recent years, hydrotalcite like compounds (i.e., layered double hydroxides, LDHs) have been widely used in the study of CO_2_ adsorption and photocatalysis, because of its unique physical and chemical properties as well as excellent catalytic properties. Recently, the use of hydrotalcite as CO_2_ adsorbent or photocatalyst has been reported in many literatures, aiming at the enhancement of adsorption and catalytic properties. For instance, Chang *et al*.^10^ found that in the Ca/Al based hydrotalcite the highly dispersed inert alumina calcium oxide coated on the surface of calcium oxide could effectively prevent the aggregation of calcium oxide particles, thereby improving the stability of adsorbent, and enhancing the hydrotalcite adsorption performance. Iguchi *et al*.^11^ prepared Al-LDHs composite by a co-precipitation method, which showed considerable photocatalytic activity for converting CO_2_ to CO. Trough co-precipitation method, hydrothermal method and roasting recombination method, Zhao *et al*.^12^ obtained a series of TiO_2_/MgAlTi-LDHs and found that the photocatalytic properties of the composite were related to the crystal shape and the crystal form of TiO_2_ loaded on hydrotalcite and the adsorption surface area.

Given the fact of low conversion and utilization of CO_2_, in our previsous study^13^, a new type of lithium aluminum hydrotalcite (Ti/Li/Al-LDHs) was developed for its utilization in efficient CO_2_ adsorption. Ti/Li/Al-LDHs with chemical composition optimized showed quite high efficiency for CO_2_ absorption and capture, and moreover the calcined Ti/Li/Al-LDHs exhibited much increased performance towards CO_2_ adsorption. As inspired by these observations, herein, a series of Ti/Li/Al-LDHs with different Ti/Li/Al molar ratios were prepared by co-precipitation method and further calcined at different temperatures. It was found that the photocatalytic activity for CO_2_ reduction was greatly on the dependence of the Ti/Li/Al molar ratios, and high temperature calcination could further increase the activity for photocatalytic conversion of CO_2_ to CH_4_. In this study, the chemical compositions were optimized and the reaction active sites were modulated to elucidate the relationship between the structure and the photocatalytic activity, which provides new ideas and theoretical guides for the further design of high efficiency photocatalyst for CO_2_ reduction.

## Results

**Figure 1:**
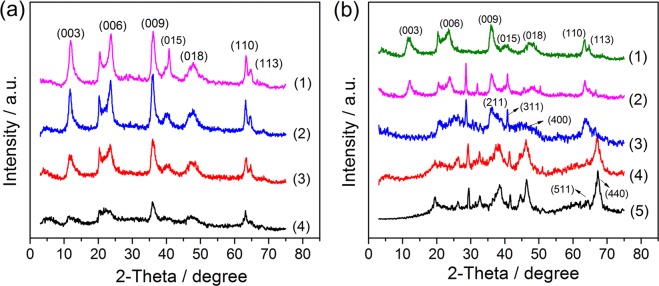
XRD patterns of (**a**) Ti/Li/Al-LDHs with different Ti:Li:Al molar ratios, (1) Ti_1_Li_3_Al_4_-LDHs, (2) Ti_1_Li_3_Al_3_-LDHs, (3) Ti_1_Li_3_Al_2_-LDHs, (4) Ti_1_Li_3_Al_1_-LDHs, and (**b**) Ti_1_Li_3_Al_2_-LDHs calcined at different temperatures (180 °C, 300 °C, 500 °C and 700 °C), (1) not calcined (Ti_1_Li_3_Al_2_-LDHs), (2) Ti_1_Li_3_Al_2_-180, (3) Ti_1_Li_3_Al_2_-300, (4) Ti_1_Li_3_Al_2_-500, (5) Ti_1_Li_3_Al_2_-700.

In this study, a series of Ti/Li/Al ternary hydrotalcites (TiLiAl-LDHs) with different Ti:Li:Al molar ratios was obtained by co-precipition method, and further calcined at different temperatures, with Ti/Li/Al molar ratios determined and summarized in Table 1. One will observe that with the increasing Al contents in the precursor solution, the Ti/Li/Al molar ratios in the obtained TiLiAl-LDHs samples could be well tuned, which display the same tendency as the Ti/Li/Al molar ratios in precursor solutions. Figure 1a shows the XRD patterns of Ti/Li/Al-LDHs with different Ti:Li:Al molar ratios. One can easily observe that all samples exhibit sharp and clear peaks corresponding to the crystal indexes of (003), (006), (009), (105), (108), (110), and (113), respectively, which are matching well with the the layered structure of LDHs. No other peaks appear, indicating the single crystalline phase for these synthesized hydrotalcite samples. It is also easily noted that the (006) peak appears a doublet peak character, which is similar to Li/Al-LDHs reported in the previous literature^14^, indicating that monovalent Li^+^ ions exist between the layers. However, because of the small quantity of Li^+^ charge, the crystal cell structure is deformed due to the uneven charge density with Li^+^ entering the laminate, leading to the splitting of the (006) peak^15^. Further comparison will find that the XRD peak intensity of Ti/Li/Al-LDHs decreases gradually with the Al^3+^ content decreasing. It is clear that the decreased contents of interlayer Al^3+^ will lead to the increased proportion of Ti^4+^. Thus, these Ti^4+^ could produce more positive charges, which cause repulsion between the neighboured layers, resulting in the deformation of crystal structures and thus the decreased crystallinity^16^.

**Table 1:** The atomic ratios of Ti:Li:Al in the precursor solution and in the obtained solid samples.

Sample	Ti_1_Li_3_Al_1_-LDHs	Ti_1_Li_3_Al_2_-LDHs	Ti_1_Li_3_Al_3_-LDHs	Ti_1_Li_3_Al_4_-LDHs
Precursor solution	1:3:1	1:3:2	1:3:3	1:3:4
Solid sample	0.98:2.12:1.13	0.99:2.37:2.14	0.96:2.62:3.10	1.02:2.72:3.96

Figure 1b shows the XRD patterns of Ti_1_Li_3_Al_2_-LDHs calcined at different temperatures. With the calcination temperatures increasing, the XRD peaks assigned to hydrotalcite show gradual decrease in peak intensity, and the the (003) and (110) diffraction peaks even disappear with the calcination temperature higher 300 °C. At the same time, some new peaks with crystal indexs of (211), (311), (400) and (440) emerge, which could could be assigned to mixed oxides such as Al_2_TiO_5_ and spinel Li_4_Ti_5_O_12_. This observation demonstrates that high temperature calcination will destroy the layer structure of hydrotalcite, giving rise to the crystal structure transformation from hydrotalcite to oxides and spinel^17,18^.

**Figure 2:**
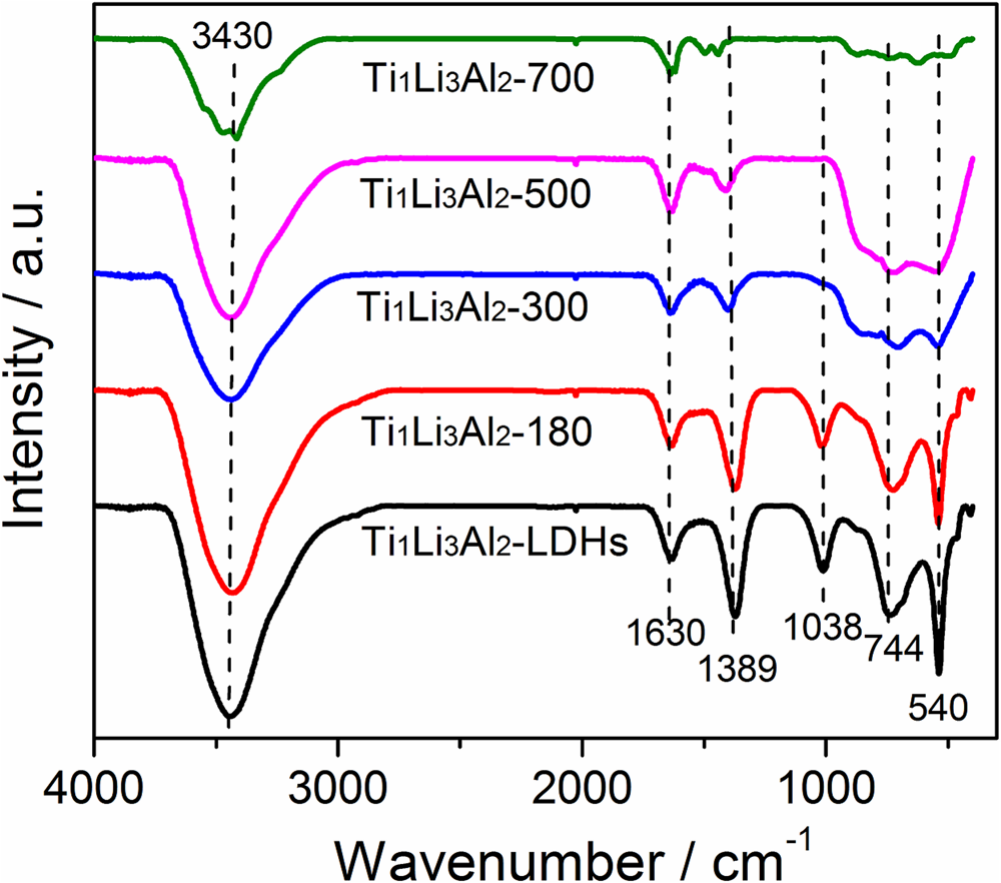
FTIR spectra of Ti_1_Li_3_Al_2_-LDHs calcined at different temperatures.

The calcination induced crystal structure transformation was further investigated by FTIR analysis. As shown in Fig. 2, both Ti_1_Li_3_Al_2_-LDHs and Ti_1_Li_3_Al_2_-T (T = 180, 300, 500 and 700) show two broad and strong absorption peaks located at ca. 3430 cm^−1^ and 1630 cm^−1^, respectively, which belong to the superposition of the hydrogen bond stretching vibration ν(O-H) absorption band of the hydroxyl group of interlayer water molecule and the bending vibration peak of O-H bond^19^. It can be seen that the hydroxyl peak decreases gradually with the increase in calcination temperatures, indicating the dehydration of the interlayer hydroxyl groups. The absorption peaks at 1389 cm^−1^ and 1038 cm^−1^, which are characteristic of the telescopic ${{\rm{CO}}}_{3}^{2-}$ carbon oxygen bond and carbon oxygen bond vibration absorption peak, respectively^20^, shows gradually weakened intensities and even disappears, implying the removal of ${{\rm{CO}}}_{3}^{2-}$ during high temperature calcination. The absorption peaks located at 540 cm^−1^ and 744 cm^−1^ should be related to the Ti-O bond and the Li-O bond^21^, both of which gradually fuse into a wide absorption peak with intensities weakened depending on the increasing calcination temperatures. All these observations suggest the destroyed layered structure of hydrotalcite and the crystal structure transformation from hydrotalcite to mixed oxides of Li_4_Ti_5_O_12_ and spinel Al_2_TiO_5_ during high temperature calcination^22^.

**Figure 3:**
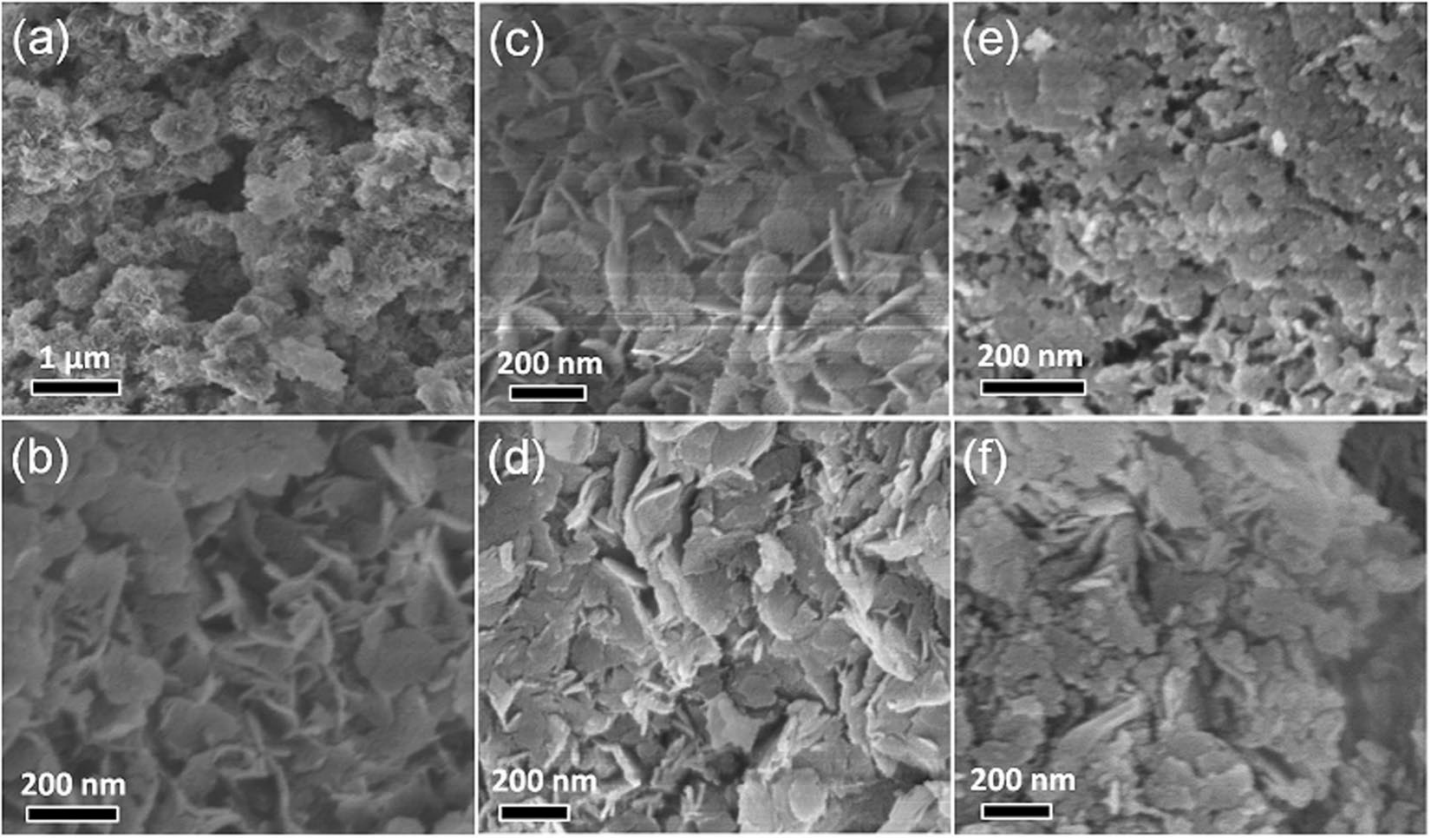
SEM images of (**a**,**b**) Ti_1_Li_3_Al_2_-LDHs and (**c**–**f**) Ti_1_Li_3_Al_2_-T, (**c**) T = 180, (**d**) T = 300, (**e**) T = 500, and (**f**) T = 700.

The morphology of Ti/Li/Al-LDHs was investigated by SEM images. With very similar morphology obtained for those Ti/Li/Al-LDHs with different Ti:Li:Al molar ratios, herein, the morphology of Ti_1_Li_3_Al_2_-LDHs was analyzed as the typical sample in details. Clearly, the Ti_1_Li_3_Al_2_-LDHs sample shows morphology in cluster-like coral reefs with serious particles agglomeration (Fig. 3a), due to the large free energy of the nanoparticles system^23^. One could also easily observe that the Ti_1_Li_3_Al_2_-LDHs particles are comprised of numerous petal-like nanosheets with thickness estimated to be tens of nanometers (Fig. 3b), which evidences the layered structure of the obtained tenary hydrotalcites. When calcined at different temperatures, the Ti_1_Li_3_Al_2_-LDHs shows a significant change in morphology, as shown in Fig. 3c–f. In comparison to Ti_1_Li_3_Al_2_-180 with layer structure well maintained (Fig. 3c), Ti_1_Li_3_Al_2_-300 shows slight deformation in the lamellar structure (Fig. 3d), and further increase in calcination temperatures brings significant morphology change to Ti_1_Li_3_Al_2_-500 and Ti_1_Li_3_Al_2_-700 with lamellar structure almost completely destroyed (Fig. 3e,f). It is well known that the increase in free energy of nanoparticle system will lead to nanoparticles agglomeration. After dehydration, Ti_1_Li_3_Al_2_-LDHs particles are more easily to agglomerate, especially after high temperature calcination, as induced by the increasing free energy of nanoparticles, which should be the main reason for the morphology change. Moreover, high temperature calcination gives rise to the crystal structure transformation from hydrotalcite to LiO, Li_4_Ti_5_O_12_, Al_2_TiO_5_ and other mixed oxides with low crystallization as supported by XRD and FTIR analysis.

**Figure 4:**
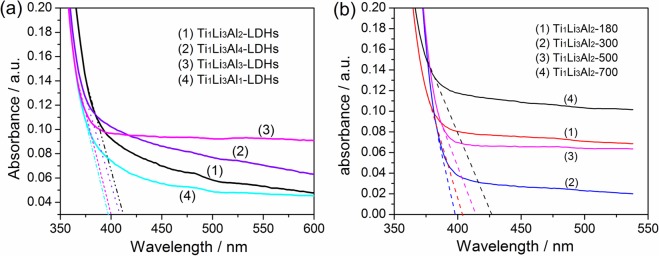
UV-Vis spectra of (**a**) Ti/Li/Al-LDHs with different Ti:Li:Al molar ratios and (**b**) Ti_1_Li_3_Al_2_-LDHs calcined at different temperatures.

The optical properties of Ti/Li/Al-LDHs with different Ti:Li:Al molar ratios were investigated by UV-vis diffuse reflectance spectra. As shown in Fig. 4a, all the Ti/Li/Al-LDHs samples show strong optical absorption in the ultraviolet light region. With optical absorption onsets (*λ*_0_) determined to be 397 nm, 413 nm, 401 nm and 408 nm, respectively, the band gaps (*E*_*g*_) could be calculated to be 3.23 eV, 3.10 eV, 3.14 eV and 3.13 eV for Ti_1_Li_3_Al_1_-LDHs, Ti_1_Li_3_Al_2_-LDHs, Ti_1_Li_3_Al_3_-LDHs and Ti_1_Li_3_Al_4_-LDHs, by the *E*_*g*_ ~ *λ*_0_ relationship (i.e., *E*_*g*_ = hc/*λ*_0_ = 1240/*λ*_0_), herein, h is the Planck constant, and c is the speed of light^24^. It is clear that with the increase of Al^3+^ content, Ti/Li/Al-LDHs shows very similar band gaps, which might not be determinative to their different photocatalytic activities. Given O 2p states mainly comprising the valence band of the hydrotalcites^25–27^, the conduction band of the Ti/Li/Al-LDHs samples should be negative enough for driving the CO_2_ reduction reaction, as deduced from their band gaps (>3.0 eV)^28^. However, all the Ti/Li/Al-LDHs samples possess band gap much smaller than the previously reported (Cu)/Zn(Fe)/Al-LDHs (about 4.10–4.50 eV)^29,30^. Thus, it could be deduced that these obtained Ti/Li/Al-LDHs could efficiently utilize ultraviolet light to excite electrons from valence band to conduction band to trigger photocatalytic conversion of CO_2_ to CH_4_. After calcined at different temperatures, the Ti_1_Li_3_Al_2_-LDHs shows obvious change in optical absorption. As shown in Fig. 4b, the Ti_1_Li_3_Al_2_-T (T = 180, 300, 500, and 700) samples show optical absorption onsets at 404 nm, 398 nm, 414 nm and 427 nm, respectively, with band gaps determined to be 3.07 eV, 3.12 eV, 3 eV and 2.90 eV. Comparative analysis demonstrates that the band gap of Ti_1_Li_3_Al_2_-T is widened first and then narrowed, depending on the increasing calcination temperature. These results imply that the conductivity of hydrotalcite decreases first and then increased, with the gradual deformation of the layered structure and the crystal structure transformation from hydrotalcite to mixed metal oxides during high temperature calcination. For instance, with the calcination temperature higher than 500 °C, the lamellar structure of Ti_1_Li_3_Al_2_-T (T = 500 and 700) collapses completely, with hydrotalcite transformed to Li_4_Ti_5_O_12_, Al_2_TiO_5_ and other spinel mixed oxides, which should give rise to the obvious red shift in optical absorption and then benefit the photocatalytic CO_2_ reduction reaction for the Ti_1_Li_3_Al_2_-700 sample as discussed in the following sections.

**Figure 5:**
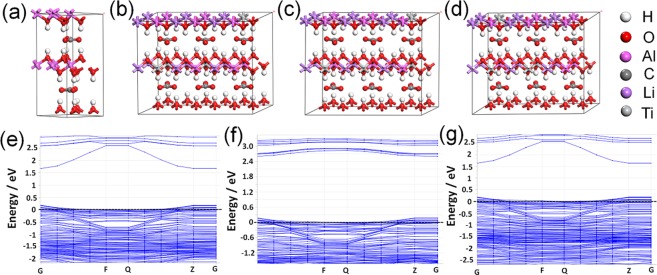
Model of (**a**) Al-LDHs, (**b**) Ti_1_Li_3_Al_2_-LDHs-(I), (**c**) Ti_1_Li_3_Al_2_-LDHs-(II), (**d**) Ti_1_Li_3_Al_2_-LDHs-(III), and The energy band diagram of (**e**) Ti_1_Li_3_Al_4_-LDHs-(І), (**f**) Ti_1_Li_3_Al_4_-LDHs-(ІI), (**g**) Ti_1_Li_3_Al_4_-LDHs-(ІII).

Given the narrowest band gap of Ti_1_Li_3_Al_2_-LDHs benefiting the photocatalytic process, the crystal structure and electronic structure of Ti_1_Li_3_Al_2_-LDHs were further optimized and calculated by Density Functional Theory^31^. As shown in Fig. 5a, the 2H model is adopted in the Al-LDHs structure and the [Al_8_(OH)_16_]_2_CO_3_ fundamental model is constructed. The ${{\rm{CO}}}_{3}^{2-}$ unit in the hcp-Al position is located in the middle of two parallel plates (The triangle formed by the three oxygen atoms on the top of the ${{\rm{CO}}}_{3}^{2-}$ is located in the interior of the three hydroxyl oxygen of the aluminum atom of the laminated plate). Based on the structure of Al-LDHs, three representative structures of Ti_1_Li_3_Al_2_-LDHs were constructed by atomic substitution, as shown in Fig. 5b–d. In all the three structures, the ${{\rm{CO}}}_{3}^{2-}$ species tends to locate in the middle of the two adjacent layers, and does not appear to come closer towards one layer. To understand the electronic structure changes caused by atomic replacement of Al with Ti and Li, the electronic structures were calculated with the three optimized structures of Ti_1_Li_3_Al_2_-LDHs-(І), Ti_1_Li_3_Al_2_-LDHs-(ІІ), and Ti_1_Li_3_Al_2_-LDHs-(ІІІ). As shown in Fig. 5e–g, with Al atoms substituted by Ti and Li atoms, the obtained Ti_1_Li_3_Al_2_-LDHs shows band gaps calculated to be 1.489 eV, 2.427 eV and 1.464 eV, for the three representative structures. Moreover, all three structures show the Fermi level across the valence band in the calculated band structures, inducating the characteristics of p-type semiconductor for Ti_1_Li_3_Al_2_-LDHs.

**Figure 6:**
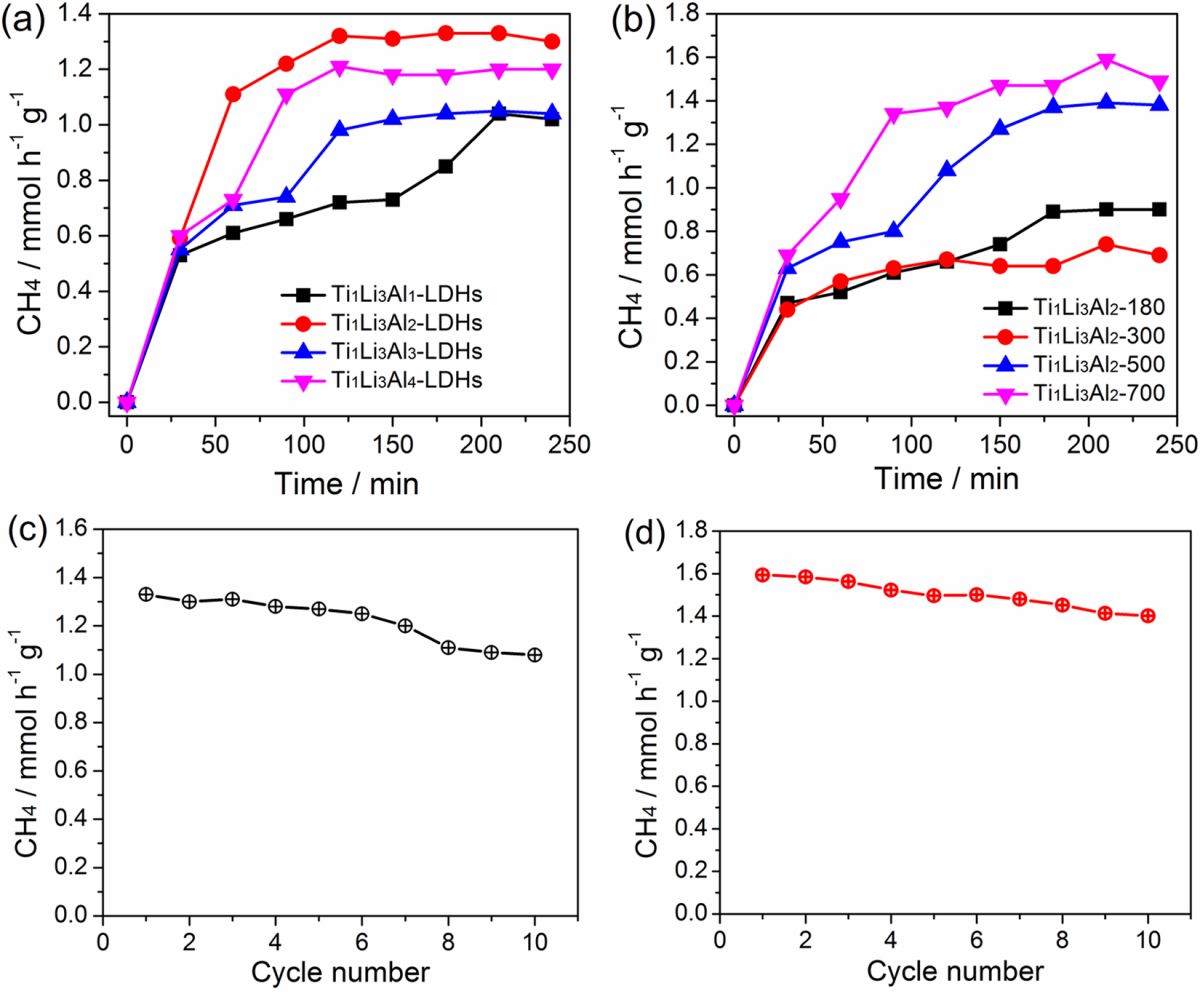
Photocatalytic CH_4_ yields of (**a**) Ti/Li/Al-LDHs with different Ti:Li:Al molar ratios, (**b**) Ti_1_Li_3_Al_2_-LDHs calcined at different temperatures, (**c**) Ti_1_Li_3_Al_2_-LDHs and (**d**) Ti_1_Li_3_Al_2_-700 during 10 reaction cycles.

The photocatalytic activity for the reduction of CO_2_ with water vapor was measured on the obtained Ti/Li/Al-LDHs under ultraviolet light irradiation. Without light irradiation or CO_2_, there is no product detected. As shown in Fig. 6a, all the Ti/Li/Al-LDHs samples display good photocatalytic activity for CO_2_ reduction with CH_4_ obtained as the main product; while other products can be hardly detected, meaning the high selectivity of CH_4_ generation from CO_2_ photoreduction. For all the samples, with the photocatalytic reaction proceeding, the CH_4_ production rates increase first and then stabilize after ca. 2 hours. This could be explained by the adequate illumination condition which is necessary to ensure the photocatalytic CO_2_ conversion reaction, and help Ti/Li/Al-LDHs generate enough electrons to drive the reaction of CO_2_ with H_2_O, producing H• and •CO_2_^−^ as the two main intermediates for CH_4_ generation^32^. It is observable that the photocatalytic activities for CO_2_ reduction is of great dependence on the Ti:Li:Al molar ratios. In comparison, the Ti_1_Li_3_Al_2_-LDHs sample shows the highest photocatalytic activity for CO_2_ reduction, with CH_4_ production rate achieving as high as 1.33 mmol h^−1^ g^−1^. As confirmed in our previous study^13^, all the obtained Ti/Li/Al-LDHs samples contain a large amount of H_2_O and ${{\rm{CO}}}_{3}^{2-}$. Moreover, the contents of H_2_O and ${{\rm{CO}}}_{3}^{2-}$ in Ti_1_Li_3_Al_2_-LDHs are the highest, which should contribute to the highest photocatalytic activity for CO_2_ reduction, given the inherent adsorption of water and ${{\rm{CO}}}_{3}^{2-}$ between hydrotalcite layers for efficient photocatalytic CO_2_ reduction reaction. The optical absorption property and the density functional calculation results suggest the characteristics of p-type semiconductor for Ti_1_Li_3_Al_2_-LDHs with the narrowest band gap among all the Ti/Li/Al-LDHs samples, which are also favorable for photocatalytic CO_2_ reduction.

With the Ti_1_Li_3_Al_2_-LDHs sample undergoing high temperature calcination, the photocatalytic CO_2_ reduction activity is decreased first and then increased, depending on the increasing calcination temperatures, as shown in Fig. 6b. The highest photocatalytic CO_2_ reduction activity is achieved over the Ti_1_Li_3_Al_2_-700 sample, with CH_4_ production rate reaching 1.59 mmol h^−1^ g^−1^. As demonstrated by the XRD, FTIR and SEM analysis, high temperature calcination will cause the deformation of the layered structure and more importantly the crystal structure transformation from hydrotalcite to mixed metal oxides such as Li_4_Ti_5_O_12_ and Al_2_TiO_5_. Especially for the samples calcined at temperatures higher than 500 °C, for instance, Ti_1_Li_3_Al_2_-700, the mixed oxides with high reactive oxygen species is generated in a large amount, which benefits the photocatalytic decomposition of H_2_O to produce active hydrogen species, activating the photocatalytic CO_2_ reduction for CH_4_ production^33^.

**Figure 7:**
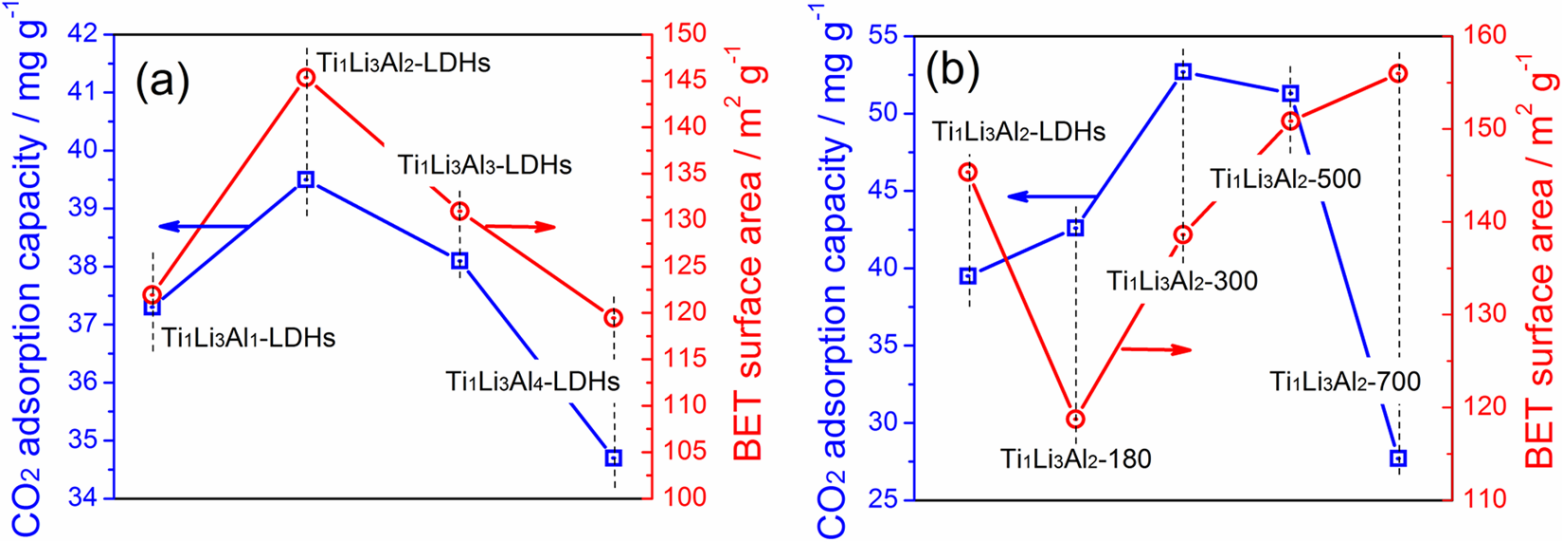
Surface area and the CO_2_ adsorption capacity of (**a**) Ti/Li/Al-LDHs with different Ti:Li:Al molar ratios, (**b**) Ti_1_Li_3_Al_2_-LDHs calcined at different temperatures.

It is well recognized that photocatalytic CO_2_ reduction process must be involved with several steps, including CO_2_ adsorption, light absorption and photoexcitation, charge separation and transfer, surface CO_2_ reduction reaction, and each step could determine the CO_2_ photoreduction activity. In this study, Ti/Li/Al-LDHs was used as photocatalyst for CO_2_ reduction. Given the almost the same band gaps of Ti/Li/Al-LDHs with different Ti:Li:Al molar ratios (Fig. 4a), light absorption and photoexcitation should not be the determinant for their different photocatalytic activities. Interestingly, as shown in Fig. 7a, with the increasing Ti:Li:Al molar ratios, both the surface area and the CO_2_ adsorption capacity reach the highest for Ti_1_Li_3_Al_2_-LDH, which corresponds well with its highest photocatalytic activity. This observation implies that the CO_2_ adsorption capacity should greatly determine the photocatalytic CO_2_ reduction activities of these obtained Ti/Li/Al-LDHs samples. For Ti_1_Li_3_Al_2_-LDHs calcined at different temperatures, depending on the increasing calcination temperatures, one can hardly find any relationship between the surface area and the CO_2_ adsorption capacity (Fig. 7b). It is somewhat surprising that Ti_1_Li_3_Al_2_-700 display the smallest CO_2_ adsorption capacity, despite of the highest surface area. More importantly, Ti_1_Li_3_Al_2_-700 shows the highest photocatalytic activity for CO_2_ reduction, which indicates that in this case some other reasons rather than the CO_2_ adsorption capacity should be decisive to the CO_2_ photoreduction activity. Given the band gap determining the light absorption ability and then the electron photoexcitation, the highest CO_2_ photoreduction activity achieved over Ti_1_Li_3_Al_2_-700 should be then mainly due to its reduced band gap haversting more light for photoexcitation (Fig. 4b).

As an excellent photocatalyst, except for the high photocatalytic activity, good stability is also highly required. Herein, the photocatalytic stabilities of Ti_1_Li_3_Al_2_-LDHs and Ti_1_Li_3_Al_2_-700 were measured during a 10-cycle photocatalytic reaction. As shown in Fig. 6c and d, both Ti_1_Li_3_Al_2_-LDHs and Ti_1_Li_3_Al_2_-700 exhibit considerable photocatalytic stability during the 10-cycle photocatalytic measurement (ca. 2500 min), with photocatalytic CH_4_ production rates decreased by only 19% and 12%, respectively. Given the almost unchanged XRD patterns of the samples after the CO_2_ photoreduction reaction (data not shown), such decrease in photocatalytic activity of Ti_1_Li_3_Al_2_-LDHs should be due to the inactivation of photoactive sites in the layered structure of hydrotalcite, while high temperature calcination will turn these active sites to high reactive oxygen species in mixed oxides, which should be more stable for photocatalytic CO_2_ reduction, ensuring the better stability of Ti_1_Li_3_Al_2_-700. Therefore, to obtain a high efficiency LDHs photocatalyst, it is highly desired to optimize the cation molar ratio and then identify the photocatalytic active sites to elucidate the photocatalytic mechanism for CO_2_ reduction with high activity, selectivity and stability.

## Discussion

In this study, a series of tenary Ti/Li/Al-LDHs with different Ti:Li:Al molar ratios was synthesized by a simple co-precipitation method, and investigated for photocatalytic CO_2_ reduction. It was found that the Ti/Li/Al-LDHs sample with Ti:Li:Al molar ratio optimized to be 1:3:2 displayed the highest photocatalytic activity for CO_2_ reduction, with CH_4_ production rate achieving 1.33 mmol h^−1^ g^−1^, which should be related to the the interlayer anion content and the narrowest band gap, as supported by both experimental and theoretical evidences. After calcined at different temperatures, the photocatalytic activity for CO_2_ reduction could be further improved. Especially, for the sample calcined at 700 °C, the photocatalytic CH_4_ production rate was increased to be 1.58 mmol h^−1^ g^−1^, and the activity was well maintained during a 10-cycle measurement. Such photocatalytic enhancement should be mainly attributed to the cystal structure transformation from hydrotalcite to mixed oxides containing high reactive oxygen species for more efficient CO_2_ reduction. This study successfully developed a facile approach to prepare multiple cations contained LDHs as efficient and stable photocatalysts for CO_2_ reduction, and demonstrated that the composition optimization and reactive site evolution could be pivotal to the high efficiency photocatalytic CO_2_ reduction.

## Methods

### Materials preparation

A series of Ti/Li/Al-LDHs was prepared by a co-precipitation method. Typically, a 200 mL of aqueous solution prepared by mixing 0.03 mol of LiCl and desired amounts of TiCl_4_ and AlCl_3_ in a flask. Under stirring, a mixed solution of KOH and NaCO_3_ was dropped into the as-prepared mixture solution in flask to ajust the pH value at 7~8. After stirred for another 1 h, the mixture solution was kept at 75 °C for 36 h, and then filtered and washed with distilled water for several times to completely remove Cl^−^ in the solution. Then Ti/Li/Al-LDHs was obtained after dried at 80 °C for 24 h. With molar ratios of Ti:Li:Al in the mixture solutions set to be 1:3:1, 1:3:2, 1:3:3, and 1:3:4, a series of Ti/Li/Al-LDHs with different Ti:Li:Al molar ratios was successfully obtained, and labeled as as Ti_1_Li_3_Al_1_-LDHs, Ti_1_Li_3_Al_2_-LDHs, Ti_1_Li_3_Al_3_-LDHs, and Ti_1_Li_3_Al_4_-LDHs, respectively.

The Ti_1_Li_3_Al_2_-LDHs sample was futher calcined for 30 min in air at different temperatures (180 °C, 300 °C, 500 °C and 700 °C), and the obtained samples were labeled as Ti_1_Li_3_Al_2_-T (T = 180, 300, 500 and 700).

### Characterizations

**Figure 8:**
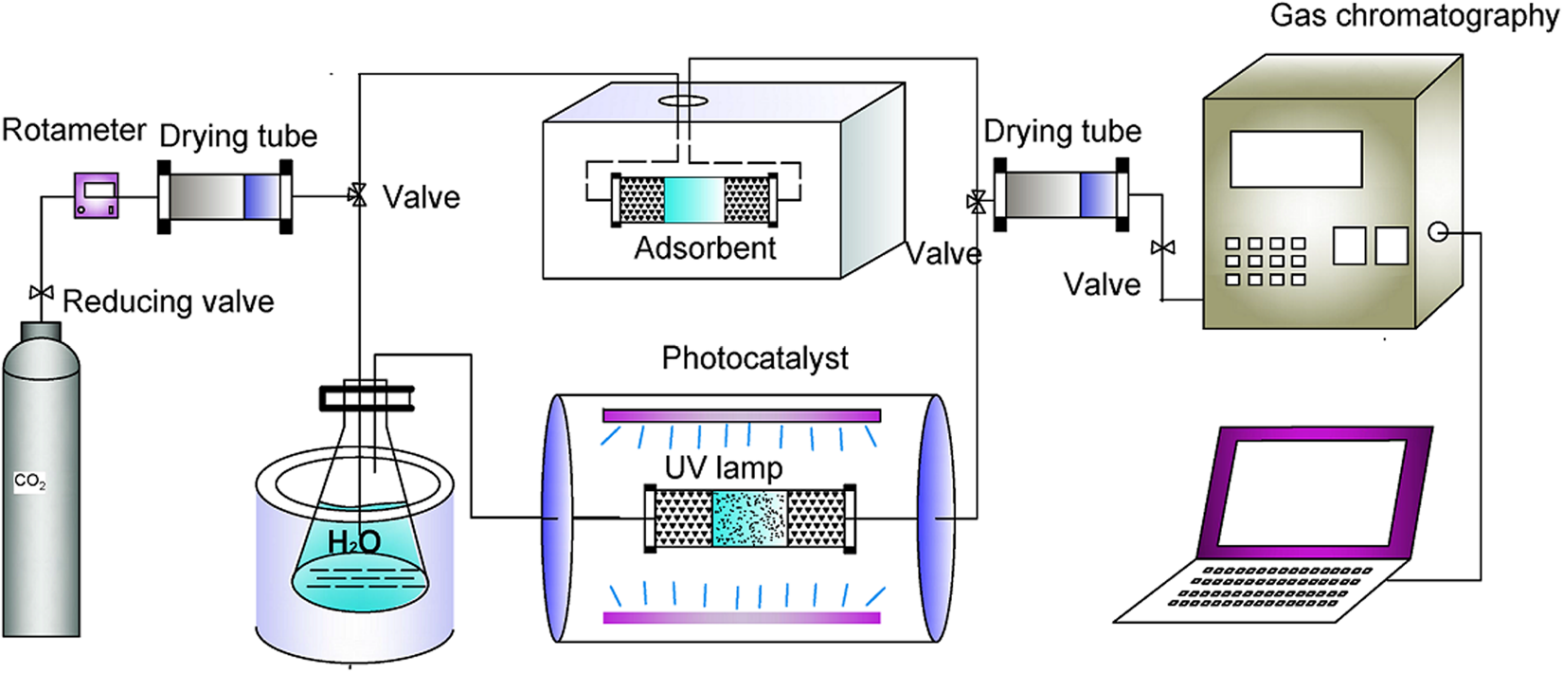
Schematic of the apparatus for CO_2_ adsorption and photocatalytic CO_2_ reduction.

The molar ratios of the samples were determined by Atomic Absorption Spectrometer (AAS Various 6, Analytik Jena AG, German). X-ray diffraction (XRD) patterns were collected on a MiniFlex600 desktop X-ray diffractometer operated at 30 kV and 10 mA using Cu Kα irradiation (Wavelength = 1.5406 Å). Scanning electron microscopy (SEM) images were recorded on a S-4800 scanning electron microscope at an accelerating voltage of 200 kV. Fourier transform infrared (FTIR) spectra were recorded on a Brook Tensor 27 Fourier transform infrared spectrometer) using the KBr pellet technique. UV-vis diffuse reflectance spectra were recorded on a Perkin Elmer Lambda 950 ultraviolet spectrophotometer. N_2_ adsorption-desorption isotherms were conducted at 77 K using an Accelerated Surface Area and Porosimetry Analyzer (ASAP2010, Micromeritics) after degassing the samples at 100 °C for 4 h. The specific surface areas were determined by the Brunauer-Emmett-Teller (BET) methods. The CO_2_ adsorption capacity was calculated from the weights of the samples before and after CO_2_ adsorption in a home-made quartz fixed bed reactor (Fig. 8).

### Computation methods

The crystal structure and electronic structure of Ti_1_Li_3_Al_2_-LDHs were optimized and calculated by Density Functional Theory. Based on the structure of Al-based hydrotalcite, the structure of Ti_1_Li_3_Al_2_-LDHs was constructed by atomic substitution, and built by Statistical Disorder included in Materials Studio. Statistical Disorder can build all possible structural models in an exhaustive way. The final structure model has 13186 structures, and each containing 144 atoms. Herein, we choose three representative structures, and calculated them in arithmetic average. Three structures are named as Ti_1_Li_3_Al_2_-LDHs-(I), Ti_1_Li_3_Al_2_-LDHs-(II), and Ti_1_Li_3_Al_2_-LDHs-(III), which will be described in Results and Discussion.

In order to predict the stable structure of hydrotalcite with specific measurement ratio, the structure is optimized. All calculations are performed using the CASTEP module in the Materials Studio package. By using Broyden-Fletcher-Goldfarb-Shanno structure optimization (BFGS) algorithm, the atomic positions and cell parameters are optimized at the same time, the convergence of standard energy is 5.0 × 10^−6^ eV·atom^−1^, the convergence criteria for each atomic force is less than 0.01 eV/Å, the displacement deviation is 5 × 10^−4^ Å, and the pressure deviation is 0.02 GPa. The exchange correlation functionals are LDA-CA-PZ, the pseudopotential uses the OTFG form of the super soft pseudopotential, and the electron minimization method uses the Pulay density mixing method (Density, Mixing). The mixed error self-consistent field calculation is 5 × 10^−7^ eV·atom^−1^, the truncation kinetic energy is 630 eV, while the Brillouin zone K vector is selected for the 5 × 5 × 2. The total charge number is 0, and the inter layer has a weak interaction, such as van Edward force.

### Photocatalytic CO_2_ reduction

The activity of photocatalytic conversion of CO_2_ to CH_4_ was tested in a continuous entry type system (Fig. 8), including a home-made quartz fixed bed reactor equipped with an on-line gas detection system (Agilent 7890 A gas chromatograph). For photocatalytic CO_2_ reduction, 1.0 g of photocatalyst was loaded in the middle part of the quartz tube reactor, with temperature heated up to 60 °C. CO_2_ gas with the flow of 80 mL/min was continuously passed through the water vapor generator and then introduced into the photocatalytic reactor. CO_2_ photocatalytic reduction was triggered under UV irradiation, with produced gas analyzed by on-line gas chromatograph with the temperature of the injector, column and detector set at 120 °C, 50 °C, 100 °C, respectively.

## References

[1] IPCC. Special Report on Renewable Energy Sources and Climate Change Mitigation, http://www.ipcc.ch/report/srren/2011.

[2] Tang LQ (2018). Zn_x_Cd_1−x_S tunable band structure-directing photocatalytic activity and selectivity of visible-light reduction of CO_2_ into liquid solar fuels. Nanotechnology.

[3] Buursink ML (2014). Significance of carbon dioxide density estimates for basin-scale storage resource assessments. Energy Proced..

[4] Bhown AS, Freeman BC (2011). Analysis and status of post-combustion carbon dioxide capture technologies. Environ. Sci. Technol..

[5] Olah GA, Goeppert A, Prakashi GKS (2009). Chemical recycling of carbon dioxide to methanol and dimethyl either. From greenhouse gas to tenewable, environmentally carbon neutral fuels and synthetic hydrocarbons. J. Org. Chem..

[6] Wang B (2018). Oxygen-Vancancy-Activated CO_2_ splitting over amorphous oxide semiconductor photocatalyst. ACS Catal..

[7] Rao H (2017). Visible-light-driven methane formation from CO_2_ with a molecular iron catalyst. Nature.

[8] Pougin A, Dilla M, Strunk J (2016). Identification and exclusion of intermediates of photocatalytic CO_2_ reduction on TiO_2_ under conditions of highest purity. Phys. Chem. Chem. Phys..

[9] Bai S, Zhang N, Gao C, Xiong Y (2018). Defect engineering in photocatalytic materials. Nano Energy.

[10] Chang P (2011). Ca-rich Ca-Al-oxide, high-temperature-stable sorbents prepared from hydrotalcite precursors: synthesis, characterization, and CO_2_ capture capacity. ChemSusChem.

[11] Iguchi S (2015). Photocatalytic conversion of CO_2_ in an aqueous solution using various kinds of layered double hydroxides. Catal. Today.

[12] Zhao H (2016). CO_2_ photoreduction with water vapor by Ti-embedded MgAl layered double hydroxides. J. CO2 Utilization.

[13] Kong TT (2016). Preparation of hydrotalcite-like Ti/Li/Al-LDHs and its performance in CO_2_. adsorption. J. Fuel Chem. Technol..

[14] Azzouz A (2013). Polyol-modified layered double hydroxides with attenuated basicity for a truly reversible capture of CO_2_. Adsorption.

[15] Shao M (2011). The synthesis of hierarchical Zn-Ti layered double hydroxide for efficient visible-light photocatalysis. Chem. Eng. J..

[16] Teruel L, Bouizi Y, Atienzar P (2010). Hydrotalcities of zinc and titanium as precursors of finely dispersed mixed oxide semiconductors for dye-sensitized solar cells. Energy Environ. Sci..

[17] Hu XM (2015). Hybrid photoanodes based on nanoporous lithium titanate nanostructures in dye-sensitized solar cells. J. Inorg. Mater..

[18] Mayra G (2017). Role of the synthesis route on the properties of hybrid LDH-graphene as basic catalysts. Appl. Surf. Sci..

[19] Ding X (2012). Hydrothermal synthsis, structural analysis and performance of regular Mn-Zn-Mg-Al-CO_3_ quaternary layered double hydroxides(LDHs). Chin. J. Inorg. Chem..

[20] Xue XY, Zhang SH, Zhang HM (2015). Structures of LDHs intercalated with ammonia and the thermal stability for ploy(vinylchloride). Am. J. Anal. Chem..

[21] Zhang Y (2015). A facile approach to superhydrophobic LiAl-layered double hydroxide film on Al-Li alloy substrate. J. Coat. Technol. Res..

[22] Xi YZ, Davis RJ (2008). Influence of water on the activity and stability of activated Mg-Al hydrotalcites for the transesterification of tributyrin with methanol. J. Catal..

[23] Zhang Z (2014). The influence of synthesis method on the CO_2_ adsorption capacity of Mg_3_Al-CO_3_ hydrotalcite-denived adsorbents. Sci. Adv. Mater..

[24] Shen S, Guo P, Zhao L, Du Y, Guo L (2011). Insights into photoluminescence property and photocatalytic activity of cubic and rhombohedral ZnIn_2_S_4_. J. Solid State Chem..

[25] Li J, Yang YJ (2018). New type ternary NiAlCe layered double hydroxide photocatalyst for efficient visible-light photoreduction of CO_2_ into CH_4_. Mater. Res. Exp..

[26] Silva CG, Bouizi Y, Fornés V, García H (2009). Layered double hydroxides as highly efficient photocatalysts for visible light oxygen generation from water. J. Am. Chem. Soc..

[27] Sahu RK, Mohanta BS, Das NN (2013). Synthesis, characterization and photocatalytic activity of mixed oxides derived from ZnAlTi ternary layered double hydroxides. J. Phys. Chem. Solids.

[28] Shen S (2018). Titanium dioxide nanostructures for photoelectrochemical applications. Prog. Mater. Sci..

[29] Sakr AAE (2013). Synthesis of Zn-Al LDHs intercalated with urea derived anions for capturing carbon dioxide from natural gas. J. Taiwan Inst. Chem. Eng..

[30] Kong TT (2016). Preparation of Cu/Fe/Al-LDHs and photocatalytic reduction of CO_2_ prepare CH_4_. J. Xi’an Univ. Sci. Technol..

[31] Segall MD (2002). First-principles simulation: ideas, illustrations and the CASTEP code. J. Phys.-Condens. Matter.

[32] Centi G, Perathoner S (2009). Opportunities and prospects in the chemical recycling of carbon dioxide to fuels. Catal. Today.

[33] Xiang G (2010). Large-scale synthesis of metastable TiO_2_(B) nanosheets with atomic thickness and their photocatalytic properties. Chem. Commun..

